# Supplementary material for: Mental toughness in the Football Association Women’s Super League: Relationships with playing experience, perceptions of club infrastructure, support mechanisms and self-esteem
Source: PLoS One. 2023 May 17;18(5):e0285594. doi: 10.1371/journal.pone.0285594 (PMC10191361; doi:10.1371/journal.pone.0285594)
Supplement: Attachment — Submitted filename: Responses to reviewers 2.docx [file pone.0285594.s001.docx]

**31^st^ March 2023**

Dear Dr Senel,

Thank you for the opportunity to revise our manuscript entitled: **Mental toughness in the Football Association Women’s Super League: Relationships with playing experience, perceptions of club infrastructure, support mechanisms and self-esteem - PONE-D-23-01152**

To accompany this response to the reviewers are two submitted documents:

1. An unmarked ‘clean’ version of the revised manuscript
2. An marked changes version highlighting where revisions have been made

Please find below, detailed comments illustrating how we have responded to the thoughtful suggestions of the reviewers.

All reviewer suggestions and comments are in yellow

All author responses are *italicized*

A full reference list is provided at the end of this document

Reviewer #1: First, I would like to commend the authors for choosing to target women’s football as their group of interest – this is a population that is sorely underrepresented in mental toughness (MT) research. Second, I have selected "major revisions" because I believe the discusion section needs work, all other comments, I believe, are "minor" in nature. I believe this manuscript has potential to be a valuable addition to the MT literature and would be happy to re-review the manuscript should the authors choose to re-submit.

*Response: Thank you for noting the importance of studies in this under-researched area. We appreciate the indication that our manuscript would make a valuable addition to the MT literature.*

Minor points:
Throughout the manuscript, there are a number of times where sentences are started with “That”, “These”, “It” and words of the like without clarification on what is being referred to.

*Response: This has been adjusted where possible.*

There is inconsistent use of the phrase “mental toughness” and the abbreviation “MT” throughout manuscript. I suggest a thorough proofread prior to re-submission.

*Response: Thank you – usage of MT is now consistent and the manuscript has been subjected to further proofreading.*

Abstract
Line 34-35. I am confused by the sentence “However, there exists only limited research on the contribution of MT to elite women’s football.” It is not clear what this means. Are you talking about MT contributing to performance? Are you talking about how MT interacts with other factors? Are you talking about MT’s relevance to the women’s form of the game?

*Response: Thank you for this recommendation. The manuscript now states:*

*“However, the extent to which MT is related to playing experiences and appreciation of club environment in elite women’s football has received only limited research attention. Accordingly, the present study investigated MT in the context of the English Football Association Women’s Super League (WSL). Specifically, this paper examined relationships between level of MT and external (playing experience, perceptions of club infrastructure, and appreciation of support mechanisms) and internal (self-esteem) factors.”*

Introduction
P3, L74. Different parentheses used.

*Response: Amended*

P3, L75. “Based on these,…” What are you referring to here?

*Response: Thank you, the manuscript now states:*

*“Based on these attributes, Loehr [5,7,8] conceptualised MT as a psychological resource that facilitated stress tolerance and maximised an individual’s capacity, regardless of circumstances, to perform consistently towards the upper range of their abilities.”*

P4, L84-86. “Some theorists…” this sentence would be stronger with a reference added.

*Response: Thank you. The manuscript has been amended accordingly:*

*“*Some theorists conceptualise non-cognitive skills as a mindset or allied beliefs that shape how individuals make sense of the world [16].”

P4, L93-95. You should change the reference here to Clough et al. (2002), as Gucciardi (2017) discusses a different conceptualisation.
Conceptual misalignment is an issue here. You mention Gucciardi’s (2017) definition, however, use a scale that is a derivative of Clough et al.’s (2002) 4/6 C’s conceptualisation. I would recommend either removing the paragraph on P5, L116-120 or changing this to reflect the 4/6 C’s conceptualisation.

*Response: Thank you for this clarification. The Gucciardi reference was used as an overview – the context here was misperceived. The manuscript has been amended to state:*

*“The notion that MT is a moderator of stressors is consistent with the construct’s overlap with components of hardiness (i.e., challenge, commitment, and control) [21].”*

I feel as though a stronger point could be made regarding the lack female representation in MT research. You could also highlight some research that has focussed on female athletic populations. Some examples are:
Madrigal, L., Wurst, K., & Gill, D. L. (2016). The role of mental toughness in coping and injury response in female roller derby and rugby athletes. Journal of Clinical Sport Psychology, 10(2), 137-154.
Wilson, D., Bennett, E. V., Mosewich, A. D., Faulkner, G. E., & Crocker, P. R. (2019). “The zipper effect”: Exploring the interrelationship of mental toughness and self-compassion among Canadian elite women athletes. Psychology of Sport and Exercise, 40, 61-70.

*Response: Thank you for the suggestion of integrating these studies and making stronger the dearth of studies of MT in female elite sporting contexts. We have addressed these comments in the manuscript:*

*“Preceding work on MT is also restricted by a limited focus on female athletes. This group in comparison to male performers are underrepresented within MT literature. Studies focusing on MT female sportspersons have produced important findings that have extended conceptual understanding of the role that MT plays in performance. For instance, Madrigal, et al.[46] , using female athletes who participated in roller derby and collegiate rugby, found MT was both related to adaptive coping and positive injury response, and engaging in activity when injured. Another example is Wilson et al. [47], who through conducting semi-structured interviews with Canadian elite women athletes, identified that MT was critical for coping with sport-related adversity. Particularly, participants experienced MT as a coping resource, reflecting perseverance, maintaining perspective, satisfactory competition preparation, and focusing/re-focus attention to the present moment. In addition to MT, common humanity, mindfulness, and self-kindness facilitated the capacity to cope with sport-related adversity. Furthermore, MT and self-compassion were perceived as compatible and contextually related processes. Self-compassion was vital to the development of mental toughness, and mindfulness was key to developing and maintaining self-compassion and MT. Findings from studies such as those conducted by Madrigal et al. [46] and Wilson et al. [47] are conceptually important because they broaden understanding of MT by providing insights into the way in which female elite athletes experience, perceive, and conceptualize MT. This is essential if sporting practitioners are to effectively use MT to enhance performance and well-being in sportspersons.”*

P10, L229. It seems odd that H1 states there will be a strong positive relationship between self and peer-rated MT, when, in the introduction, you mention Wieser and Thiel’s study finding no agreement between player and coach ratings. If this is because you are focussed on peer-ratings? If so, you may need to mention this.

*Response: Thank you. We have added research reporting positive relationships between self and other ratings and changed the emphasis within H1*:

*“Regarding triangulation of perceptions of MT, Gucciardi et al. [44], with a sample of Australian football players, observed no significant group differences between self, parent, and coach ratings. However, correlational analyses revealed only low to moderate relationships among raters. This is outcome was consistent with research in other contexts (e.g., organisational) where self vs. other rater agreements have demonstrated low to moderate, yet positive, relationships [45].*

Methods
I really like the employment of a peer-assessor of MT. I believe it is a simple step that should be adopted as widely as possible in the MT literature in cross-sectional designs. Well done, authors.

P14, L345-347. Good idea to have similar positions assess MT.

*Response: Thank you for noting the methodological rigour behind our assessment of MT*

I was unable find details on what program (e.g., SPSS, R) was used to run the analysis.

*Response: We have included two references to the statistical program in the revised manuscript.*

Results
It may be useful to clarify which MT scores you included in the moderation analysis (i.e., the self-report or peer-reported). Only because you mention how peer-assessment may provide more accurate assessment of attitudes and behaviours in the discussion.

*Response: We have added that analyses were performed in relation to self-rated scores.*

Discussion
The discussion needs work. As it stands it is too vague and needs to incoporate more research and theory - this will add more punch to your conclusions. For instance, the paragraph beginning on P22, L523. It appears the main message is that MT may influence how one views their club infrastructure – which is an important finding and adds to the collective understanding of MT. However, vague suggestions for future research are offered instead of discussing how this finding exists within our current understanding of MT. You could have referred to Nicholls et al. (2016) who linked MT to perceptions of coaching, or discussed Meggs and Chen (2019) who explored MT and attribution style (these are just examples). I think adopting this approach will strengthen the discussion and highlight your findings more clearly. I recommend trying to integrate more MT research into each section of your discussion where you mention a key finding.
Refs:
Meggs, J., & Chen, M. A. (2018). Mental toughness and attributions of failure in high performing male and female swimmers. Journal of Human Sport and Exercise, 13(2), 276-284.
Nicholls, A. R., Morley, D., & Perry, J. L. (2016). Mentally tough athletes are more aware of unsupportive coaching behaviours: Perceptions of coach behaviour, motivational climate, and mental toughness in sport. International Journal of Sports Science & Coaching, 11(2), 172-181.

*Response: Thank you for this suggestion. A significant revision of the discussion of the manuscript has been undertaken. The two references suggested above and others have been integrated. The perception of club infrastructure has been brought to the fore. The most significant of the changes made are below:*

*“There were no significant relationships between MT and Head Coach Leadership, Internal Support, and Learning Culture. A possible way to cultivate interactions between infrastructure and MT is to consider the contributory role of coaching style and climate, which previous research have identified as important factors [82].* *Nicholls, Morley, & Perry [82] found that supportive coach behaviours were positively associated with task-involving climates, which in turn was positively associated with MT. The term supportive coaching refers to a broad, multifaceted coaching style that incorporates discrete yet interrelated emotional/relational and structural/instrumental components of effective instruction. The use of supportive coaching is important because the approach guides goal striving and nurtures development of athletic and mental skills.*

*In this context, supportive behaviours enhance athlete’s self-efficacy, problem-solving, and ability to cope with stress [83]. The findings of Nicolas, Gaudreau, & Franche [83] indicated that this process is positively allied to MT through a task-involving climate. That is, a sporting environment where athletes perceive the focus of training to be skill mastery. The combination of supportive coach behaviours and task-involving climate create a setting in which effort and improvement are recognised and rewarded. Applied to the present study, this suggests that club infrastructure will increase levels of MT when coaches provide a reassuring, enabling environment that focuses on core skills and positively acknowledges endeavour and development. Reciprocally, positive perceptions of coaching style and purpose should heighten player appreciation of club infrastructure.*

*Another important factor that influences engagement with infrastructure is players’ attributional styles. Meggs and Chen [84] found that high MT scores, within high performing swimmers, were positively associated with controllability. Specifically, possession of an attributional style, which viewed sporting failure as the result of internal, manageable factors. While self-perceived controllability is beneficial to the extent that it helps athletes maintain self-belief and perseverance, from a club perspective it may be problematic since individuals high in MT will generally be more independent and only seek support during times of transition and critical incidents [74]. This suggests that they are most likely to draw on club infrastructure when it is pertinent to their personal needs. Hence, sporting organisations, where possible, should attempt to create an accommodating infrastructure that aligns club facilities with individual demands [85**]. One way to achieve this is to provide general services that are accessed via assessment of specific player needs. A concomitant advantage may be that players relate more strongly with the club because they see close alignment between personal and club identity. This is an important connection to nurture as the between club and player association can have a significant influence on performance and individual well-being [86].*

*The failure to find relationships between MT and infrastructure (Head Coach Leadership, and Learning Culture) and Internal Work Support, was contrary to the notion that players with high MT were more likely to constructively appraise these factors because they offer opportunities for self-development, innovation, and challenge. However, the findings of the present study should be treated cautiously due to the fact that provision varied greatly across clubs. Moreover, differences between club infrastructures were potentially exacerbated by unanticipated constraints (i.e., changes in club management and disruptions arising from COVID-19). Hence, future investigations should determine the extent to which player evaluations of head coach leadership, learning culture, and internal support are affected by variables such as the degree to which structures are embedded within the organisation, changes in team management, length of time players have been at a club, duration of playing career, and level of seniority. Establishing this will enable an informed assessment of the potential role that MT plays in player engagement with organisational facilities.*

*Indeed, it is important to understand interrelationships between MT, external (playing experience, perceptions of club infrastructure, and appreciation of support mechanisms) and internal (self-esteem) factors because these variables collectively indicate which types of provision within sporting organisations are likely to maximise the psychological well-being and contentment of players. Additionally, the potential importance of infrastructure was demonstrated by large positive correlations between Learning Culture and Head Coach Leadership and Internal Work Support. These suggest that constructive appraisal of core club infrastructure elements are interrelated. Acknowledging the interrelatedness of internal organisational elements, preceding research should examine interactions further and examine how MT influences associations.”*

P19, 439-440. Re-word sentence. Perhaps replace “specifies” with “demonstrates.”
P19, L443-444. It is not clear what this sentence means.

*Response: Thank you for these suggestions. The manuscript has been significantly rewritten and now states:*

*“From a psychometric perspective, consensus between rating* *scores and MTQ10 scores supported the construct validity of MT [69]. Notwithstanding this outcome, subsequent research should further explore the utility of MT peer ratings. This could include using multiple fellow players and coaches/managers. Triangulation between ratings would demonstrate commonality and stability across sporting viewpoints. It would also further assess whether the differences observed between players and coaches in preceding work extended to coaches/managers working within elite female football.”*

P19, 460-461. It is not clear what this sentence means.

*Response: The section has been changed significantly. Thank you for the suggestion*

*“The finding that length and level of involvement in football was associated with higher MT and Self-Esteem aligned with previous sports-related studies that have identified experience as a central factor allied to MT (e.g., football [48] and cricket [35]). It concurred also with the supposition that relations were due to increased knowledge [3]. Explicitly, learning how to deal with difficulties, experiencing failure, and encountering demanding environments [74].”*

P22, L513-522. May be useful to mention Gucciardi et al. (2017). Particularly as it was study with an all female cohort.

*Response: Thank you, this reference is now included.*

P22, L523. It’s not clear why you have started this sentence with “intuitively.” Also, how might these attributes affect assessment of club infrastructure?

*Response: As part of the significant rewrite of the discussion, this section has now been adjusted and extended:*

*“Hence, future investigations should determine the extent to which player evaluations of head coach leadership, learning culture, and internal support are affected by variables such as the degree to which structures are embedded within the organisation, changes in team management, length of time players have been at a club, duration of playing career, and level of seniority. Establishing this will enable an informed assessment of the potential role that MT plays in player engagement with organisational facilities.*

*Indeed, it is important to understand interrelationships between MT, external (playing experience, perceptions of club infrastructure, and appreciation of support mechanisms) and internal (self-esteem) factors because these variables collectively indicate which types of provision within sporting organisations are likely to maximise the psychological well-being and contentment of players. Additionally, the potential importance of infrastructure was demonstrated by large positive correlations between Learning Culture and Head Coach Leadership and Internal Work Support. These suggest that constructive appraisal of core club infrastructure elements are interrelated. Acknowledging the interrelatedness of internal organisational elements, preceding research should examine interactions further and examine how MT influences associations.”*

P24, L572-573. Does this need to be said? Weren’t all players who took part female?

*Response: Thank you for highlighting how this sentence could be clearer. The manuscript has been changed:*

*“In the present study although the sample size was relatively small, it still included a high percentage of the female players across the WSL; there are currently only 12 teams in the WSL with squad sizes of 23.”*

Paragraph P25 L600. How might have COVID-19 impacted these findings?

*Response: Thank you for this suggestion. We address this question in two areas in the manuscript:*

*“One obvious concern is sample size, which was lower than expected due to the outbreak of the COVID-19 pandemic and ensuing restrictions. This greatly impacted upon recruitment since the principal investigators were not able to meet with teams to cultivate engagement and concomitantly several clubs experienced re-organisations meaning that commitment to involvement faltered”*

*AND*

*“Another potential limitation is that outcomes may be contextually restricted. This is a major concern since data collection occurred during the height of the COVID restrictions. Consequently, there were great disruptions to club infrastructure. This in part may explain the relationship between MT and External Support, and account for the lack of association between MT and internal measures. This is something that ensuing investigations should establish. Certainly, COVID measures provided a unique set of circumstances that may have influenced player responses. That stated, contextual effects are a common concern within psychological research and care is always required to ensure that outcomes are representative and generalised appropriately.”*

Reviewer #2: Thank you for the opportunity to review this manuscript. The authors demonstrated the concept and related factors of "Mental Toughness" in WSL athletes. The study is well conducted and the employed methods are appropriate. Regarding the description of the manuscript in the paper, there were no excesses or deficiencies in the explanations of each variable in the background. I was concerned that the results included a description of the analysis methods, but if the journal allows it, I don’t feel that it is a problem. Also, since the paper consists of many research results, it would be easier to read if the current results were summarized in the first paragraph of the Discussion or the Conclusion. It will be of interest to readers of this journal, particularly researchers and practitioners involved in the sports community.

*Response: Thank you for the kind comments regarding the design and deployment of our study. We hope that researchers and practitioners in the sports community will find this research useful. We have made a number of substantial additions to the manuscript that have improved how we have integrated this study with the extant literature. In our rewriting we have also tried to more clearly ‘signpost’ the findings in the discussion to aid interpretation*

Thank you for the opportunity to provide a revision of our manuscript

The authors

References:

1. Wrack S. Barclays unveiled as Women’s Super League sponsor in ground-breaking deal. The Guardian. 2019 March [Cited 2020 July 18]*.* Available from: https://www.theguardian.com/football /2019/mar/20/barclays-womens-super-league- sponsor-record-deal-uk-fa

2. Currie J. Emma Mitchell: Arsenal defender opens up on mental health*.* BBC Sport. 2019 October [Cited 2020 May 22]. Available from:

[https://www.bbc.co.uk/sport/football/49934256](https://www.bbc.co.uk/sport/football/49934256 3)

[3](https://www.bbc.co.uk/sport/football/49934256 3). Liew GC, Kuan G, Chin NS, Hashim HA. Mental toughness in sport. German Journal of Exercise and Sport Research. 2019 Dec 1;49(4):381-394. https://doi.org/10.1007/s12662-019-00603-3

4. Aryanto DB, Larasati A. Factors influencing mental toughness. In 5th ASEAN Conference on Psychology, Counselling, and Humanities (ACPCH 2019) 2020 Jan 22 (pp. 307-309). Atlantis Press. [https://doi.org/10.2991/assehr.k.200120.066](https://doi.org/10.2991/assehr.k.200120.066%20was%203)

5. Loehr JE. Athletic excellence: Mental toughness training for sports. Forum publishing company; 1985.

6. Luszki WA. Winning tennis through mental toughness. Everest House Publ.; 1982.

7. Loehr JE. Mental toughness training for sports: Achieving athletic excellence. Lexington, MA: Stephen Greene Press; 1986.

8. Loehr JE. The New Toughness Training for Sports: Mental Emotional Physical Conditioning from 1 World's Premier Sports Psychologist. Plume; 1995.

9. Crust L. A review and conceptual re-examination of mental toughness: Implications for future researchers. Personality and Individual Differences. 2008; 45(7): 576–583. https://doi.org/10.1016/j.paid.2008.07.005

10. Perry JL, Strycharczyk D, Dagnall N, Denovan A, Papageorgiou KA, Clough PJ. Dimensionality of the mental toughness questionnaire (MTQ48). Frontiers in Psychology. 2021 Jul 23;12:654836. https://doi.org/10.3389/fpsyg.2021.654836

11. Gucciardi D, Gordon S, Dimmock J. Advancing mental toughness research and theory using personal construct psychology. International Review of Sport and Exercise Psychology. 2009; 2(1): 54–72. https://doi.org/10.1080/17509840802705938

12. Drinkwater K, Dagnall N, Denovan A, Parker A. The moderating effect of mental toughness: perception of risk and belief in the paranormal. Psychological Reports. 2019; 122(1): 268–287. https://doi.org/10.1177/0033294118756600

13. Ren T, Yu X, Yang W. Do cognitive and non-cognitive abilities mediate the relationship between air pollution exposure and mental health? PLOS ONE. 2019; 14(10): e0223353. https://doi.org/10.1371/journal.pone.0223353

14. Borghans L, Duckworth AL, Heckman JJ, Ter Weel B. The economics and psychology of cognitive and non-cognitive traits. Journal of Human Resources. 2008; 43(4): 972–1059. https://doi.org/10.1353/jhr.2008.0017

15. Denovan A, Dagnall N, Drinkwater K. Examining what Mental Toughness, Ego Resiliency, Self-efficacy, and Grit measure: An exploratory structural equation modelling bifactor approach. Current Psychology. 2022; 1–16. <https://doi.org/10.1007/s12144-022-03314-5>

16. Gabrieli, C., Ansel, D., & Krachman, S. B. (2015). Ready to Be Counted: The Research Case for Education Policy Action on Non-Cognitive Skills. A Working Paper. Transforming Education.

17. Nicholls AR, Levy AR, Polman RCJ, Crust L. Mental toughness, coping self-efficacy, and coping effectiveness among athletes. International Journal of Sport Psychology. 2011; 42(6): 513–524.

18. Zalewska AM, Krzywosz-Rynkiewicz B, Clough PJ, Dagnall N. Mental toughness development through adolescence: Effects of age group and community size. Social Behavior and Personality: An International Journal. 2019; 47(1): 1–8. <https://doi.org/10.2224/sbp.7376>

19. Mojtahedi D, Dagnall N, Denovan A, Clough P, Hull S, Canning D, Lilley C, Papageorgiou KA. The relationship between mental toughness, job loss, and mental health issues during the COVID-19 pandemic. Frontiers in Psychiatry. 2021; 11: 607246. https://doi.org/10.3389/fpsyt.2020.607246

20. Papageorgiou KA, Denovan A, Dagnall N. The positive effect of narcissism on depressive symptoms through mental toughness: Narcissism may be a dark trait but it does help with seeing the world less grey. European Psychiatry. 2019; 55: 74–79. <https://doi.org/10.1016/j.eurpsy.2018.10.002>

21. Clough, P. J., Earle, K., & Sewell, D. (2002) Mental Toughness: The Concept and Its Measurement. In I. Cockerill (Ed.), Solutions in Sport Psychology (pp. 32-43). London: Thomson.

22. Eschleman KJ, Bowling NA, Alarcon GM. A meta-analytic examination of hardiness. International Journal of Stress Management. 2010; 17(4): 277–307. <https://doi.org/10.1037/a0020476>

23. Kobasa SC. Stressful life events, personality, and health: an inquiry into hardiness. Journal of Personality and Social Psychology. 1979; 37(1): 1–11. https://doi.org/10.1037/0022-3514.37.1.1

24. Maddi SR. The story of hardiness: Twenty years of theorizing, research, and practice. Consulting Psychology Journal: Practice and Research. 2002; 54(3): 173–185. https://doi.org/10.1037/1061-4087.54.3.173

25. Nowack K, Niemirowski A. Cognitive hardiness in coaching: Personality trait, skill, or outcome. Coaching Psychologist. 2021; 17(1):14-31. https://doi.org/10.53841/bpstcp.2021.17.1.14

26. Gucciardi DF, Gordon S, Dimmock JA. Towards an understanding of mental toughness in Australian football. Journal of Applied Sport Psychology. 2008; 20(3): 261–281. <https://doi.org/10.1080/10413200801998556>

27. Meggs J, Ditzfeld C, Golby J. Self-concept organisation and mental toughness in sport. Journal of Sports Sciences. 2014; 32(2): 101–109. https://doi.org/10.1080/02640414.2013.812230

28. Kruger E. Mental toughness is a predictor of suicidality in university students. Education and Health. 2018; 36(2): 42–47.

29. Marchant DC, Polman RC, Clough PJ, Jackson JG, Levy AR, Nicholls AR. Mental toughness: managerial and age differences. Journal of Managerial Psychology, 2009; 24(5): 428–437. https://doi.org/10.1108/02683940910959753

30. Clough P, Oakes S, Dagnall N, St Clair-Thompson H, McGeown S. The study of non-cognitive attributes in education: Proposing the mental toughness framework. In Non-cognitive skills and factors in educational attainment. Brill; 2016. pp. 315-329. . https://doi.org/10.1007/978-94-6300-591-3_14

31. Rusk RD, Waters LE. Tracing the size, reach, impact, and breadth of positive psychology. The Journal of Positive Psychology. 2013; 8(3): 207–221. https://doi.org/10.1080/17439760.2013.777766

32. Lin Y, Mutz J, Clough PJ, Papageorgiou KA. Mental toughness and individual differences in learning, educational and work performance, psychological well-being, and personality: A systematic review. Frontiers in Psychology. 2017; 8: 1345. <https://doi.org/10.3389/fpsyg.2017.01345>

33. Gucciardi DF. Mental toughness: progress and prospects. Current Opinion in Psychology. 2017; 16: 17–23. https://doi.org/10.1016/j.copsyc.2017.03.010

34. Gucciardi DF. Commentary: Mental toughness and individual differences in learning, educational and work performance, psychological well-being, and personality: A systematic review. Frontiers in Psychology. 2018; 8: 2329. https://doi.org/10.3389/fpsyg.2017.02329

35. Bull SJ, Shambrook CJ, James W, Brooks JE. Towards an understanding of mental toughness in elite English cricketers. Journal of Applied Sport Psychology. 2005; 17(3): 209–227. https://doi.org/10.1080/10413200591010085

36. Connaughton D, Wadey R, Hanton S, Jones G. The development and maintenance of mental toughness: Perceptions of elite performers. Journal of Sports Sciences. 2008; 26(1): 83–95. https://doi.org/10.1080/02640410701310958

37. Nicholls AR, Polman RC, Levy AR, Backhouse SH. Mental toughness, optimism, pessimism, and coping among athletes. Personality and Individual Differences. 2008; 44(5): 1182–1192. https://doi.org/10.1016/j.paid.2007.11.011

38. Sheard M, Golby J, Van Wersch A. Progress toward construct validation of the Sports Mental Toughness Questionnaire (SMTQ). European Journal of Psychological Assessment. 2009; 25(3): 186–193. https://doi.org/10.1027/1015-5759.25.3.186

39. Gucciardi DF. Do developmental differences in mental toughness exist between specialized and invested Australian footballers? Personality and Individual Differences. 2009; 47(8): 985–989. https://doi.org/10.1016/j.paid.2009.08.001

40. Golby J, Sheard M. Mental toughness and hardiness at different levels of rugby league. Personality and Individual Differences. 2004; 37(5): 933-942. https://doi.org/10.1016/j.paid.2003.10.015

41. Sheard M. A cross-national analysis of mental toughness and hardiness in elite university rugby league teams. Perceptual and Motor Skills. 2009; 109(1): 213–223. https://doi.org/10.2466/pms.109.1.213-223

41. Kelly GA. The psychology of personal constructs: A theory of personality. London: Routledge; 1991. https://doi.org/10.4324/9780203359037

42. Crust L, Nesti M, Littlewood M. A cross-sectional analysis of mental toughness in a professional football academy. Athletic Insight Journal. 2010; 2(2).

43. Wieser R, Thiel H. A survey of “mental hardiness” and “mental toughness” in professional male football players. Chiropractic & Manual Therapies. 2014; 22(1): 1–6. <https://doi.org/10.1186/2045-709X-22-17>

44. Gucciardi DF, Gordon S, Dimmock JA. Development and preliminary validation of a mental toughness inventory for Australian football. Psychology of Sport and Exercise. 2009; 10(1): 201-209. https://doi.org/10.1016/j.psychsport.2008.07.011.

45. Dierdorff EC, Surface EA. Placing peer ratings in context: Systematic influences beyond ratee performance. Personnel Psychology. 2007; 60(1): 93-126. <https://doi.org/10.1111/j.1744-6570.2007.00066.x>

46. Madrigal L, Wurst K, Gill DL. The role of mental toughness in coping and injury response in female roller derby and rugby athletes. Journal of Clinical Sport Psychology. 2016 Jun 1;10(2):137-154. https://doi.org/10.1123/JCSP.2015-0021

47. Wilson D, Bennett EV, Mosewich AD, Faulkner GE, Crocker PR. “The zipper effect”: Exploring the interrelationship of mental toughness and self-compassion among Canadian elite women athletes. Psychology of Sport and Exercise. 2019 Jan 1;40:61-70. https://doi.org/10.1016/j.psychsport.2018.09.006

48. Kristjánsdóttir H, Jóhannsdóttir KR, Pic M, Saavedra JM. Psychological characteristics in women football players: Skills, mental toughness, and anxiety. Scandinavian Journal of Psychology. 2019; 60(6): 609–615. https://doi.org/10.1111/sjop.12571

49. Williams KT, Coopoo Y, Van Niekerk RL, Fortuin CS. Mental toughness of first division women football players in Gauteng province, South Africa. African Journal for Physical Activity and Health Sciences (AJPHES). 2020; 26(4): 468-480. https://doi.org/10.37597/ajphes.2020.26.4.9

50. Eubank MR, Nesti MS, Littlewood MA. A culturally informed approach to mental toughness development in high performance sport. International Journal of Sport Psychology. 2017; 48(3): 206–222. https://doi.org/10.7352/IJSP.2017.48.206

51. Malecki CK, Demaray MK. What type of support do they need? Investigating student adjustment as related to emotional, informational, appraisal, and instrumental support. School Psychology Quarterly. 2003;18(3): 231-252. https://doi.org/10.1521/scpq.18.3.231.22576

52. Barker ET, Bornstein MH. Global self-esteem, appearance satisfaction, and self-reported dieting in early adolescence. The Journal of Early Adolescence. 2010; 30(2): 205–224. https://doi.org/10.1177/0272431609332936

53. Ciarrochi J, Heaven PC, Davies F. The impact of hope, self-esteem, and attributional style on adolescents’ school grades and emotional well-being: A longitudinal study. Journal of Research in Personality. 2007; 41(6): 1161–1178. https://doi.org/10.1016/j.jrp.2007.02.001

54. Richman EL, Shaffer DR. “IF YOU LET ME PLAY SPORTS” How Might Sport Participation Influence the Self‐Esteem of Adolescent Females? Psychology of Women Quarterly. 2000; 24(2): 189-199. https://doi.org/10.1111/j.1471-6402.2000.tb00200.x

55. Dagnall N, Denovan A, Papageorgiou KA, Clough PJ, Parker A, Drinkwater KG. Psychometric assessment of shortened Mental Toughness Questionnaires (MTQ): factor structure of the MTQ-18 and the MTQ-10. Frontiers in Psychology. 2019; 10: 1933. https://doi.org/10.3389/fpsyg.2019.01933

56. Collins NM, Cromartie F, Butler S, Bae J. Effects of early sport participation on self-esteem and happiness. The Sport Journal. 2018; 20: 1-20.

57. Zeiger JS, Zeiger RS. Mental toughness latent profiles in endurance athletes. PLOS ONE. 2018; 13(2): e0193071. https://doi.org/10.1371/journal.pone.0193071

58. Papageorgiou KA, Malanchini M, Denovan A, Clough PJ, Shakeshaft N, Schofield K, Kovas Y. Longitudinal associations between narcissism, mental toughness and school achievement. Personality and Individual Differences. 2018; 131: 105–110. https://doi.org/10.1016/j.paid.2018.04.024

59. Carless SA, Wearing AJ, Mann L. A short measure of transformational leadership. Journal of Business and Psychology. 2000; 14(3): 389–405. https://doi.org/10.1023/A:1022991115523

60. Morgeson FP, Humphrey SE. The Work Design Questionnaire (WDQ): Developing and validating a comprehensive measure for assessing job design and the nature of work. Journal of Applied Psychology. 2006; 91(6): 1321–1339. https://doi.org/10.1037/0021-9010.91.6.1321

61. Singer SJ, Moore SC, Meterko M, Williams S. Development of a short-form learning organization survey: The LOS-27. Medical Care Research and Review. 2012; 69(4): 432–459. https://doi.org/10.1177/1077558712448135

62. Kliem S, Mößle T, Rehbein F, Hellmann DF, Zenger M, Brähler E. A brief form of the Perceived Social Support Questionnaire (F-SozU) was developed, validated, and standardized. Journal of Clinical Epidemiology. 2015; 68(5): 551–562. https://doi.org/10.1016/j.jclinepi.2014.11.003

63. Harris MA, Donnellan MB, Trzesniewski KH. The Lifespan Self-Esteem Scale: Initial validation of a new measure of global self-esteem. Journal of Personality Assessment. 2018; 100(1): 84–95. https://doi.org/10.1080/00223891.2016.1278380

64. Krishnaveni R, Deepa R. Controlling common method variance while measuring the impact of emotional intelligence on well-being. Vikalpa. 2013; 38(1): 41–48. https://doi.org/10.1177/0256090920130104

65. Tabachnick BG, Fidell LS, Ullman JB. Using Multivariate Statistics. Boston, MA: Pearson; 2007.

66. Efron B, Tibshirani R. Bootstrap methods for standard errors, confidence intervals, and other measures of statistical accuracy. Statistical Science. 1986; 1(1): 54–77. https://doi.org/10.1214/ss/1177013815

67. Hayes AF. Introduction to Mediation, Moderation and Conditional Process Analysis. Guilford Press; 2013.

68. Paulhus DL, Vazire S. The self-report method. Handbook of research methods in personality psychology. 2007;1(2007):224-239.

69. Paunonen SV, O'Neill TA. Self‐reports, peer ratings and construct validity. European Journal of Personality: Published for the European Association of Personality Psychology. 2010; 24(3): 189–206. <https://doi.org/10.1002/per.751>

70. Williams C, Denovan A, Drinkwater K, Dagnall N. Thinking style and paranormal belief: the role of cognitive biases. Imagination, Cognition and Personality. 2022 Mar;41(3):274-298. https://doi.org/10.1177/02762366211036435

71. Dagnall N, Denovan A, Parker A, Drinkwater K, Walsh RS. Confirmatory factor analysis of the inventory of personality organization-reality testing subscale. Frontiers in Psychology. 2018 Jul 5;9:1116. https://doi.org/10.3389/fpsyg.2018.01116

72. Bensch D, Maaß U, Greiff S, Horstmann KT, Ziegler M. The nature of faking: A homogeneous and predictable construct?. Psychological Assessment. 2019 Apr;31(4):532-544. https://doi.org/10.1037/pas0000619

73. Gignac GE, Szodorai ET. Effect size guidelines for individual differences researchers. Personality and Individual Differences. 2016; 102:74-78. https://doi.org/10.1016/j.paid.2016.06.069

74. Crust L, Clough PJ. Developing mental toughness: From research to practice. Journal of Sport Psychology in Action. 2011 Apr 1;2(1):21-32. https://doi.org/10.1080/21520704.2011.563436

75. Anstiss PA, Meijen C, Marcora SM. The sources of self-efficacy in experienced and competitive endurance athletes. International Journal of Sport and Exercise Psychology. 2010; 18(5): 622–638. https://doi.org/10.1080/1612197X.2018.1549584

76. Brace AW, George K, Lovell GP. Mental toughness and self-efficacy of elite ultra-marathon runners. PLOS ONE. 2020; 15(11): e0241284. <https://doi.org/10.1371/journal.pone.0241284>

77. Podlog L, Eklund RC. A longitudinal investigation of competitive athletes' return to sport following serious injury. Journal of Applied Sport Psychology. 2006 Mar 1;18(1):44-68. https://doi.org/10.1080/10413200500471319

78. Rosenberg M, Schooler C, Schoenbach C, Rosenberg F. Global self-esteem and specific self-esteem: Different concepts, different outcomes. American Sociological Review. 1995; 141–156. https://doi.org/10.2307/2096350

79. Petrie TA, Deiters J, Harmison RJ. Mental toughness, social support, and athletic identity: Moderators of the life stress–injury relationship in collegiate football players. Sport, Exercise, and Performance Psychology. 2014; 3(1): 13–27. <https://doi.org/10.1037/a0032698>

80. Cobb, S. (1976). Social support as a moderator of life stress. Psychosomatic medicine, 38(5), 300-314. https://doi.org/10.1097/00006842-197609000-00003

81. Lane AM, Jones L, Stevens MJ. Coping with failure: The effects of self-esteem and coping on changes in self-efficacy. Journal of Sport Behavior. 2002 Dec 1;25(4):331-346.

82. Nicholls AR, Morley D, Perry JL. Mentally tough athletes are more aware of unsupportive coaching behaviours: Perceptions of coach behaviour, motivational climate, and mental toughness in sport. International Journal of Sports Science & Coaching. 2016 Apr;11(2):172-181. https://doi.org/10.1177/1747954116636714

83. Nicolas M, Gaudreau P, Franche V. Perception of coaching behaviors, coping, and achievement in a sport competition. Journal of Sport and Exercise Psychology. 2011 Jun 1;33(3):460-468. https://doi.org/10.1123/jsep.33.3.460

84. Meggs J, Chen MA. Mental toughness and attributions of failure in high performing male and female swimmers. Journal of Human Sport and Exercise. 2018 Mar 20;13(2):276-284. https://doi.org/10.14198/jhse.2018.132.03

85. Eubank MR, Nesti MS, Littlewood MA. A culturally informed approach to mental toughness development in high performance sport. International Journal of Sport Psychology. 2017 May 1;48(3):206-222.

86. Tibbert SJ, Andersen MB, Morris T. What a difference a “Mentally Toughening” year makes: The acculturation of a rookie. Psychology of Sport and Exercise. 2015 Mar 1;17:68-78. https://doi.org/10.1016/j.psychsport.2014.10.007

87. McAuley AB, Hughes DC, Tsaprouni LG, Varley I, Suraci B, Baker J, Herbert AJ, Kelly AL. Genetic associations with personality and mental toughness profiles of English academy football players: An exploratory study. Psychology of Sport and Exercise. 2022: 61: 102209. https://doi.org/10.1016/j.psychsport.2022.102209

88. Farnsworth JL, Marshal A, Myers NL. Mental toughness measures: A systematic review of measurement properties for practitioners. Journal of Applied Sport Psychology. 2021; 34(3): 479–494. https://doi.org/10.1080/10413200.2020.1866710

89. Dagnall N, Drinkwater KG, Denovan A, Walsh S. The potential benefits of non-skills training (mental toughness) for elite athletes: Coping with the negative psychological effects of the COVID-19 pandemic. Frontiers in Sports and Active Living. 2021; 3:581431. https://doi.org/10.3389/fspor.2021.581431
